# How representative are neuroimaging samples? Large-scale evidence for trait anxiety differences between fMRI and behaviour-only research participants

**DOI:** 10.1093/scan/nsab057

**Published:** 2021-05-05

**Authors:** Caroline J Charpentier, Paul Faulkner, Eva R Pool, Verena Ly, Marieke S Tollenaar, Lisa M Kluen, Aniek Fransen, Yumeya Yamamori, Níall Lally, Anahit Mkrtchian, Vincent Valton, Quentin J M Huys, Ioannis Sarigiannidis, Kelly A Morrow, Valentina Krenz, Felix Kalbe, Anna Cremer, Gundula Zerbes, Franziska M Kausche, Nadine Wanke, Alessio Giarrizzo, Erdem Pulcu, Susannah Murphy, Alexander Kaltenboeck, Michael Browning, Lynn K Paul, Roshan Cools, Karin Roelofs, Luiz Pessoa, Catherine J Harmer, Henry W Chase, Christian Grillon, Lars Schwabe, Jonathan P Roiser, Oliver J Robinson, John P O’Doherty

**Affiliations:** Division of Humanities and Social Sciences, California Institute of Technology, Pasadena, CA 91125, USA; Institute of Cognitive Neuroscience, University College London, London WC1N 3AZ, UK; Department of Psychology, University of Roehampton, London SW15 5PJ, UK; Department of Psychology, University of Geneva, Geneva 1205, Switzerland; Department of Clinical Psychology, Leiden University, Leiden, 2333 AK, The Netherlands; Leiden Institute for Brain and Cognition, Leiden University, Leiden 2300 RC, The Netherlands; Department of Clinical Psychology, Leiden University, Leiden, 2333 AK, The Netherlands; Leiden Institute for Brain and Cognition, Leiden University, Leiden 2300 RC, The Netherlands; Division of Humanities and Social Sciences, California Institute of Technology, Pasadena, CA 91125, USA; Division of Humanities and Social Sciences, California Institute of Technology, Pasadena, CA 91125, USA; Institute of Cognitive Neuroscience, University College London, London WC1N 3AZ, UK; Institute of Cognitive Neuroscience, University College London, London WC1N 3AZ, UK; Institute of Cognitive Neuroscience, University College London, London WC1N 3AZ, UK; Institute of Cognitive Neuroscience, University College London, London WC1N 3AZ, UK; Institute of Cognitive Neuroscience, University College London, London WC1N 3AZ, UK; Institute of Cognitive Neuroscience, University College London, London WC1N 3AZ, UK; Department of Psychology, University of Maryland, College Park, MD 20742, USA; Department of Cognitive Psychology, Universität, Hamburg 20146, Germany; Department of Cognitive Psychology, Universität, Hamburg 20146, Germany; Department of Cognitive Psychology, Universität, Hamburg 20146, Germany; Department of Cognitive Psychology, Universität, Hamburg 20146, Germany; Department of Cognitive Psychology, Universität, Hamburg 20146, Germany; Department of Cognitive Psychology, Universität, Hamburg 20146, Germany; Department of Psychology, University of Geneva, Geneva 1205, Switzerland; Department of Psychiatry, University of Oxford, Oxford OX3 7JX, UK; Department of Psychiatry, University of Oxford, Oxford OX3 7JX, UK; Oxford Health NHS Foundation Trust, Warneford Hospital, Oxford OX4 4XN, UK; Department of Psychiatry, University of Oxford, Oxford OX3 7JX, UK; Department of Psychiatry and Psychotherapy, Clinical Division of Social Psychiatry, Medical University of Vienna, Vienna 1090, Austria; Department of Psychiatry, University of Oxford, Oxford OX3 7JX, UK; Oxford Health NHS Foundation Trust, Warneford Hospital, Oxford OX4 4XN, UK; Division of Humanities and Social Sciences, California Institute of Technology, Pasadena, CA 91125, USA; Donders Institute for Brain, Cognition and Behaviour, Radboud University, Nijmegen, 6525 AJ, The Netherlands; Department of Psychiatry, Radboud University Medical Centre, Nijmegen 6500 HB, The Netherlands; Donders Institute for Brain, Cognition and Behaviour, Radboud University, Nijmegen, 6525 AJ, The Netherlands; Department of Psychology, University of Maryland, College Park, MD 20742, USA; Department of Psychiatry, University of Oxford, Oxford OX3 7JX, UK; Oxford Health NHS Foundation Trust, Warneford Hospital, Oxford OX4 4XN, UK; Department of Psychiatry, University of Pittsburgh, Pittsburgh, PA 15213, USA; National Institute of Mental Health, Bethesda, MD 20892, USA; Department of Cognitive Psychology, Universität, Hamburg 20146, Germany; Institute of Cognitive Neuroscience, University College London, London WC1N 3AZ, UK; Institute of Cognitive Neuroscience, University College London, London WC1N 3AZ, UK; Division of Humanities and Social Sciences, California Institute of Technology, Pasadena, CA 91125, USA

**Keywords:** trait anxiety, neuroimaging, behaviour, sampling bias

## Abstract

Over the past three decades, functional magnetic resonance imaging (fMRI) has become crucial to study how cognitive processes are implemented in the human brain. However, the question of whether participants recruited into fMRI studies differ from participants recruited into other study contexts has received little to no attention. This is particularly pertinent when effects fail to generalize across study contexts: for example, a behavioural effect discovered in a non-imaging context not replicating in a neuroimaging environment. Here, we tested the hypothesis, motivated by preliminary findings (*N* = 272), that fMRI participants differ from behaviour-only participants on one fundamental individual difference variable: trait anxiety. Analysing trait anxiety scores and possible confounding variables from healthy volunteers across multiple institutions (*N* = 3317), we found robust support for lower trait anxiety in fMRI study participants, consistent with a sampling or self-selection bias. The bias was larger in studies that relied on phone screening (compared with full in-person psychiatric screening), recruited at least partly from convenience samples (compared with community samples), and in pharmacology studies. Our findings highlight the need for surveying trait anxiety at recruitment and for appropriate screening procedures or sampling strategies to mitigate this bias.

## Introduction

Neuroimaging methods, such as functional magnetic resonance imaging (fMRI), have been fundamental to the emergence of cognitive neuroscience as a research field. These methods provide a unique window into the function of the human brain and into the implementation of cognitive processes at the computational, neural and network levels. However, a key question that has not been examined in the field is whether individuals who participate in fMRI studies differ from those who participate in behaviour-only studies in terms of their psychological or psychiatric profiles. Given that many studies in cognitive neuroscience involve a behavioural piloting phase to assess behavioural effects, followed by an fMRI scanning phase to assess neural mechanisms, it is important to ensure that individuals who volunteer to participate in each study context exhibit similar profiles and can be characterized by similar population distributions. This is especially relevant for studies in which effects that are present (and replicate) outside the scanner (Bolton and Robinson, 2017) fail to replicate (Garibbo *et al.*, 2019) inside the scanning environment. Similarly, some effects may be more easily found in fMRI than in behavioural studies, due to higher alertness and/or stress associated with the scanner environment. While there is evidence that physical characteristics of the scanning environment, such as acoustic noise (Hommel *et al.*, 2012; Skouras *et al.*, 2013; Kobald *et al.*, 2016), can affect cognitive and affective processes, their neural basis and hormonal responses (Gossett *et al.*, 2018), poor generalizability across testing contexts could also be due, in part, to unanticipated biases in study recruitment.

Specifically, anxiety is likely to be a key factor influencing individuals’ decisions to select themselves into specific studies, situations or environments. Here, we formally test the hypothesis that, because of this selection bias or because of variability in screening procedures, individuals who participate in fMRI studies exhibit lower trait anxiety than individuals who participate in behavioural studies. Within populations of healthy volunteers, it is likely that anxious individuals are more nervous about going into the MRI scanner and are discouraged or excluded from participating if claustrophobic (Meléndez and McCrank, 1993; Katz *et al.*, 1994; Murphy and Brunberg, 1997). While perhaps not unexpected, the hypothesis of lower trait anxiety in fMRI study contexts has to our knowledge never been formally tested, nor do we know the extent to which the distribution of anxiety levels is likely to be reduced to a narrower range.

In addition, this question is also particularly pertinent for studies in which a modulatory effect of anxiety on behaviour is expected and for researchers interested in the mechanisms of anxiety *per se*. While anxiety disorders constitute a major global health burden (Beddington *et al.*, 2008), anxiety is also a normative adaptive function that varies across the general population. Studying anxiety in healthy human subjects can thus help bridge the gap between animal models of anxiety and clinical applications for patients with anxiety disorders (Grillon *et al.*, 2019; Robinson *et al.*, 2019). Myriad studies have suggested that a wide range of cognitive functions are modulated by anxiety levels (see Robinson *et al.*, 2013, for a review): sensory processing and gating (Grillon, 2002; Engel-Yeger and Dunn, 2011; Poli and Angrilli, 2015), attentional biases towards negative emotional stimuli (Bar-Haim *et al.*, 2007; Cisler and Koster, 2010), decreased emotion regulation (Etkin *et al.*, 2010; Farmer and Kashdan, 2012), deficits in attentional control (Bishop, 2009), reduced working memory performance (Shackman *et al.*, 2006; Yao *et al.*, 2018), impairments during reinforcement learning (Browning *et al.*, 2015; Mkrtchian *et al.*, 2017) and increased risk avoidance during decision-making (Maner *et al.*, 2007; Clark *et al.*, 2012; Charpentier *et al.*, 2017). Neuroimaging studies have provided evidence for heightened amygdala responses to negative emotional stimuli (Etkin *et al.*, 2004; Stein *et al.*, 2007) and reduced connectivity between the prefrontal cortex and the amygdala (Etkin *et al.*, 2010; Robinson *et al.*, 2014; Shackman *et al.*, 2016; Carlisi and Robinson, 2018) in anxiety. Because of this multifaceted association between anxiety and cognition, many behavioural and neuroimaging studies in cognitive neuroscience routinely collect measures of anxiety. A common self-report measure of anxiety can be obtained from the State-Trait Anxiety Inventory (STAI) (Spielberger *et al.*, 1983). Trait anxiety scores from the STAI range from 20 to 80, with higher scores indicating higher general proneness to anxiety. Normative data (Knight *et al.*, 1983; Spielberger *et al.*, 1983) suggest that most people from a healthy population score between 20 and 50 (mean score around 35), while scores above 50 may indicate some clinical relevance for an anxiety disorder (Fisher and Durham, 1999; Kennedy *et al.*, 2001; Julian, 2011).

If healthy volunteers who participate in fMRI studies exhibit lower anxiety levels than the general population, this could constrain the generalizability of fMRI data and have important implications for studies investigating processes associated with anxiety more specifically. For example, associations between brain responses and anxiety levels in healthy volunteers may not extend to the full range of anxiety scores typically observed in the general population. When applied to clinical studies, in-scanner effect sizes for differences between clinically anxious patients and controls may be overestimated, due to controls being abnormally ‘low’ in anxiety compared with the average population estimate.

Initial support for our hypothesis of lower trait anxiety in fMRI study participants arose from preliminary pilot and published data from three studies (Charpentier *et al.*, 2016a,b, 2018). Results from this preliminary data set are summarized in Table 1. Trait anxiety was indeed lower in the fMRI study context than in the behavioural study context (*T*_270_ = 2.679, *P* = 0.01, Cohen’s *d* = 0.384). There was no gender or age difference between study contexts, meaning those factors were unlikely to drive the observed difference in trait anxiety. However, the sample size (*N* = 272) was small (especially for the MRI context), and one factor that could be driving the difference in trait anxiety is whether participants were appropriately screened for psychiatric/affective disorders. In this preliminary sample, all fMRI subjects were screened, while a large proportion of the behaviour subjects (*N* = 145 out of 208) were not. In addition, all these data were collected by one experimenter at one institution, making it difficult to generalize.

**Table 1.** Summary of preliminary data set (*N* = 272)

			Study context difference		
Preliminary data	fMRI	Behaviour	Statistic	*P*-value	Effect size
*N*	64	208			
Gender: *N*_F_/*N*_M_	33/31	117/91	χ^2^ = 0.435	0.51	0.080
Trait anxiety (±s.d.)	34.422 (±8.44)	38.226 (±10.35)	*T*_270_ = 2.679	0.01	0.384
Age (±s.d.)	25.891 (±5.76)	24.995 (±7.65)	*T*_270_ = 0.864	0.39	0.124

Notes: Independent, two-sample *t*-tests were run assuming unequal variance. Effect sizes for *t*-tests are Cohen’s *d* values, and effect sizes for chi-square tests are standardized mean difference effect sizes calculated with the *esc_chisq* function in R. For both types of effect sizes, 0.2 is considered a small effect, 0.5 a medium effect and 0.8 a large effect.

Therefore, we set out to gather a large data set of existing trait anxiety scores from behavioural and fMRI studies involving healthy volunteers across multiple institutions. In order to account for possible confounds and examine interaction effects, we additionally collected the following variables: gender, age, whether and how participants were screened for affective/psychiatric disorders, whether the study involved the presence of a stressor and/or pharmacological manipulation, whether the study was part of anxiety research, the type of sample recruited, study duration, compensation rate and whether the data were provided before or after participant exclusion.

## Methods

### Procedure

Trait anxiety total scores, from the STAI (Spielberger *et al.*, 1983), were obtained for a total of 3317 healthy adult participants (18 years and older) across nine study sites and five countries: California Institute of Technology (USA), University of Maryland (USA), National Institute of Mental Health (USA), Universität Hamburg (Germany), Radboud University (the Netherlands), Leiden University (the Netherlands), University College London (UK), University of Oxford (UK) and University of Geneva (Switzerland). These excluded data from the preliminary data set. A summary of the final data set is provided in Table 2. Only data that were previously collected in the different contributing labs were gathered, and data were completely de-identified before sharing. Possible duplicates—trait anxiety scores from the same participant in several different studies from the same lab—cannot be identified and are therefore not accounted for, although we expect the number of duplicates to be negligible. We asked labs to provide the following information along with trait anxiety scores: gender, age (in years), whether the study was a behavioural-only study or involved functional MRI scanning (study context), whether participants were appropriately screened for affective/psychiatric disorders (see Supplementary Table S2 for details of screening procedure), whether the study involved the presence of a stressor and/or drug administration, whether recruitment was from a community or convenience sample, whether data was provided before or after exclusions, study duration, pay rate, and a short description of the study. The project was approved by the Caltech Institutional Review Board (minimal risk and exempt decision).

**Table 2.** Summary of final data set (*N* = 3317)

			Study context difference		
Final data	fMRI (*N* = 1341)	Behaviour (*N* = 1976)	Statistic	*P*-value	Effect size
Gender: % female	51.2	55.5	χ^2^ = 5.76	0.016	0.083
Trait anxiety	35.772 (±8.31)	37.820 (±9.98)	*T*_3180_ = 6.41	<0.0001	0.219
Age	24.135 (±5.85)	25.638 (±7.45)	*T*_3176.9_ = 6.40	<0.0001	0.220
Screening: % yes	64.7	63.2	χ^2^ = 0.852	0.36	0.032
Stressor: % yes	50.3	39.2	χ^2^ = 40.48	<0.0001	0.222
Drug: % yes	13.9	25.8	χ^2^ = 68.68	<0.0001	0.291
Sample: % community	21.4	27.2	χ^2^ = 480.9	<0.0001	0.824
% convenience	68.8	33.2			
Anxiety research: % yes	13.4	24.7	χ^2^ = 63.63	<0.0001	0.280
All subjects included: % yes	88.2	69.4	χ^2^ = 160.01	<0.0001	0.450
Study duration (min)	176.9 (±131.5)	176.3 (±174.6)	*T*_3279.4_ = 0.106	0.92	0.004
Pay rate in USD/h	25.97 (±22.5)	17.25 (±15.2)	*T*_2164.3_ = 12.42	<0.0001	0.472

Notes: For continuous variables, the table indicates mean values for each study context (±s.d.) and results from independent, two-sample *t*-tests (assuming unequal variance). For discrete variables, percentages are shown. Trait anxiety, age and gender were obtained for each individual; the other variables display study-level characteristics. Effect sizes for *t*-tests are Cohen’s *d* values, and effect sizes for chi-square tests are standardized mean difference effect sizes calculated with the *esc_chisq* function in R. For both types of effect sizes, 0.2 is considered a small effect, 0.5 a medium effect and 0.8 a large effect.

### Data analysis—mixed effect models

Using the *lme4* package in R (Bates *et al.*, 2015), two mixed effects models were built (i) to examine the effect of study context (behaviour *vs* fMRI) while competing for variance with the other variables (Model 1) and (ii) to assess interaction between group and other variables (Model 2). Model 1 included fixed effects of study context, gender, age, psychiatric screening, stressor, drug administration, sample type, study duration, pay rate, whether the study was part of anxiety research, and whether data were provided after participant exclusion, as well as a fixed intercept and a random intercept (grouped by study site). Model 2 included the same effects as Model 1, with the addition of a random effect of study context (grouped by study site) and the following fixed interaction effects: context × gender, context × age, context × psychiatric screening, context × stressor, context × drug administration, context * sample type, context * study duration, and context * pay rate. Study site was included as a random factor in all analyses, given the variability in mean trait anxiety across study sites (Table 3, all data column). For both models, subjects with missing gender or age data (*N* = 103) were excluded, and for Model 2, subjects from study sites that only provided data for one study context (*N* = 173) were excluded to allow for the estimation of a random effect of condition for each study site. Model 1 thus included data from 3214 subjects, and Model 2 data included data from 3041 subjects. To determine the significance of individual effects, nested model comparison was performed, using chi-square test in R to compare the full model with the corresponding model lacking the one effect of interest. The ‘anova’ function was used to compute analysis of variance tables for model comparisons. Effect sizes were obtained for pairwise comparisons of the marginal means using the *eff_size* function from the *emmeans* package in R.

**Table 3.** Data summary by study site

	All data		fMRI		Behaviour		Difference		
Study site	*N*	Trait anxiety (±s.d.)	*N*	Trait anxiety (±s.d.)	*N*	Trait anxiety (±s.d.)	*t*	*P*	Effect size (*d*)
Site #1	255	38.35 (±11.19)	155	36.08 (±10.16)	100	41.85 (±11.84)	4.01	<0.001	0.53
Site #2	102	43.38 (±10.91)	0	–	102	43.38 (±10.91)	–	–	–
Site #3	890	36.12 (±7.90)	465	34.68 (±7.75)	425	37.69 (±7.78)	5.79	<0.001	0.39
Site #4	71	34.31 (±7.35)	0	–	71	34.31 (±7.35)	–	–	–
Site #5	100	35.95 (±8.13)	45	35.31 (±7.88)	55	36.47 (±8.37)	0.71	0.48	0.14
Site #6	440	39.30 (±6.95)	413	39.26 (±6.96)	27	39.81 (±6.93)	0.40	0.69	0.08
Site #7	94	29.66 (±5.71)	61	29.02 (±5.32)	33	30.85 (±6.29)	1.42	0.16	0.32
Site #8	441	34.28 (±9.40)	55	33.84 (±9.56)	386	34.34 (±9.39)	0.37	0.72	0.053
Site #9	924	38.02 (±10.53)	147	32.78 (±8.12)	777	39.01 (±10.65)	8.09	<0.001	0.61

Notes: Sample sizes and mean trait anxiety scores (±s.d.) are reported for each site, for all data and separately for the fMRI and behavioural study contexts. Statistics for the difference between fMRI and behaviour contexts are also reported in the right-most column, specifically *t* and *P*-values from two-tailed independent sample *t*-tests (unequal variance) and effect size using Cohen’s *d*.

### Data analysis—Bayesian statistics

Bayesian analyses were conducted using JASP (Love *et al.*, 2015) in order to provide support for the effects obtained with mixed effects models. Bayesian Analysis of Covariance (ANCOVA) (Rouder and Morey, 2012; Rouder *et al.*, 2012) was used with trait anxiety as a dependent variable; study context, gender, psychiatric screening, stressor and drug administration, sample type, anxiety research, and exclusion as fixed factors; age, study duration and pay rate as covariates and study site as a random factor. To mirror the mixed effect analyses, two types of Bayesian model comparisons were performed. First, we compared pairs of models either including or not including a fixed effect of interest, with all other fixed effects included—this allowed determining the significance of main effects. Second, we compared pairs of models either including or not including an interaction effect of interest, with all fixed effects and all other interactions included. Note that only interactions with study context were considered. JASP’s default prior was used. This pairwise model comparison allows drawing inference about which model best explains the data. In practice, the test generates a Bayes Factor (BF_10_), which represents the evidence for the full model relative to the null model (which here simply lacks one effect of interest). The magnitude of BF_10_ was used to interpret the strength of evidence in favour of either model (Kass and Raftery, 1995; Jarosz and Wiley, 2014; Lee and Wagenmakers, 2014; Quintana and Williams, 2018). Evidence in favour of the model of interest was considered anecdotal (1 < BF_10_ < 3), substantial (3 < BF_10_ < 10), strong (10 < BF_10_ < 30), very strong (30 < BF_10_ < 100) or decisive (BF_10_ > 100). Similarly, evidence in favour of the null model could also be qualified as anecdotal (0.33 < BF_10_ < 1), substantial (0.1 < BF_10_ < 0.33), strong (0.033 < BF_10_ < 0.1), very strong (0.01 < BF_10_ < 0.033) or decisive (BF_10_ < 0.01).

### Follow-up analyses: effect of screening procedures

To examine the role of specific psychiatric screening procedures in modulating trait anxiety differences between fMRI and behavioural study contexts, we repeated the analyses described above (mixed effect models and Bayesian tests), taking into account whether screening was performed by phone or in-person structured interview. The detailed screening procedures for each study site and study context are reported in Supplementary Table S2. We also explored the distribution of trait anxiety scores for each type of screening procedure (no screening, phone screening, or full screening) and each study context, quantifying the mean and standard deviation (Table 4) as well as the mode and 80th percentile (Figure 4) to characterize the distributions.

**Table 4.** Trait anxiety across study contexts and screening procedures

		Behaviour		fMRI		Difference		
Screening type	Site	*N*	Trait anxiety (±s.d.)	*N*	Trait anxiety (±s.d.)	*t*	*P*	*d*
No screening	All	***728***	***38.68 (±11.08)***	***473***	***38.99 (±7.30)***	***−0.58***	***0.56***	***0.031***
	#5	55	36.47 (±8.37)	45	35.31 (±7.88)	0.71	0.48	0.14
	#6	27	39.81 (±6.93)	413	39.26 (±6.96)	0.40	0.69	0.079
	#8	168	35.21 (±10.62)	15	42.40 (±10.99)	−2.51	0.01	0.68
Phone	All	***786***	***39.33 (±9.31)***	***525***	***34.36 (±7.78)***	***10.46***	***<0.001***	***0.57***
	#3	425	37.69 (±7.78)	465	34.68 (±7.75)	5.78	<0.01	0.39
	#9	260	44.13 (±9.90)	60	31.92 (±7.72)	8.94	<0.01	1.28
Full	All	***462***	***33.90 (±8.09)***	***343***	***33.50 (±9.04)***	***0.64***	***0.52***	***0.047***
	#7	33	30.85 (±6.29)	61	29.02 (±5.32)	1.49	0.14	0.33
	#8	188	33.80 (±7.93)	40	30.63 (±6.67)	2.36	0.02	0.41
	#9	241	34.39 (±8.36)	87	33.37 (±8.38)	0.97	0.33	0.12

Notes: The number of individuals, as well as mean trait anxiety and standard deviation, are shown separately for each screening procedure (no screening, phone screening and full in-person screening) and each study context (behaviour and fMRI). Numbers in bold and italics are for the entire data set, collapsing across all study sites. The breakdown for the specific sites in which the same procedure was used for both study contexts is also shown.

### Follow-up analyses: effect of state anxiety

To assess whether the difference in trait anxiety observed between fMRI and behavioural studies could in fact be explained by a difference in state anxiety, we obtained state anxiety scores (STAI-S) for a subset of participants (*N* = 2324) from the main data set. We ran a mixed level model on this subset of the data examining the effect of both study context and state anxiety (competing for variance) on trait anxiety. The model contained fixed effects of study context and state anxiety, as well as a random intercept for study site, and was compared with the same model excluding the fixed effect of the study context.

### Data and code availability

Data and code are available on the following github repository: https://github.com/ccharpen/Trait_anxiety_MRI_BH, covered under a CC-BY-4.0 licence.

## Results

### Data set summary and descriptive statistics

**Fig. 1.**
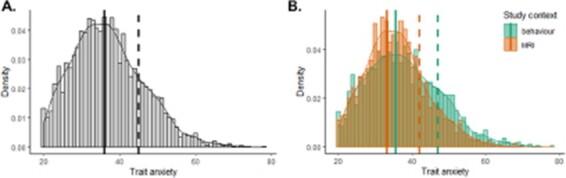
Distribution of trait anxiety scores. Density plots are shown, representing the proportion of the population at each trait anxiety score (bin = 1). Solid lines show the mode of the distribution; dashed lines the 80th percentile. (A) Distribution for the entire population (*N* = 3317): mode = 36.02, 80th percentile = 45. (B) Separate distributions for behaviour (*N* = 1976, green) and fMRI (*N* = 1341, orange) study contexts, showing both lower mode (MRI = 33.18, behaviour = 35.68) and lower 80th percentile (MRI = 42; behaviour = 47) in the fMRI study context.

The distribution of trait anxiety scores is shown in Figure 1, across the entire sample (Figure 1A) and separately for individuals participating in fMRI and behavioural studies (Figure 1B). Note that the data only pertain to healthy volunteers and do not include any clinical samples. The mean trait anxiety across the entire sample was 36.99 (±9.40), consistent with normative data (Knight *et al.*, 1983; Spielberger *et al.*, 1983). Confirming our hypothesis and preliminary data, the difference in trait anxiety between fMRI and behavioural studies was also significant in the larger sample, albeit with a smaller, but non-negligible, effect size (*t*-test assuming unequal variance: *T*_3180_ = 6.41, *P* < 0.0001; Cohen’s *d* = 0.219; Table 2). Interestingly, the distribution of trait anxiety scores across the two study contexts (Figure 1B) indicates that the difference is driven by a larger proportion of individuals in fMRI studies scoring between 30 and 40, and a larger proportion of individuals in behavioural studies scoring above 45. While the difference in mean trait anxiety between study contexts was around 2 points on the trait anxiety scale, this difference rose to 5 points when examining the 80th percentile of the distribution. According to the standard scores provided in the scale manual (Spielberger *et al.*, 1983), this 5-point difference suggests that the distribution of trait anxiety scores in fMRI studies is truncated by about 0.5 s.d. compared with that in behavioural studies.

As observed in the preliminary data, it is possible that the difference in trait anxiety could be driven by one or several of the following factors, most of them found to be significantly different between study contexts (see Table 2 for statistical inference). In the behaviour compared with fMRI context, participants were slightly older, pay rate was lower and there was a higher proportion of female participants. More fMRI studies involved the presence of a stressor, recruited from convenience samples and provided trait anxiety data including all subjects (rather than only analysed subjects), whereas more behavioural studies involved drug administration and were part of anxiety research. However, the proportion of individuals that were clinically screened was not statistically different across study contexts, nor was the average study duration. Nonetheless, we performed follow-up analyses to regress out the variance explained by these possible confounds.

### Difference in trait anxiety between fMRI and behavioural studies is robust to potential confounds

Two analyses were performed to assess the effect of study context on trait anxiety while regressing out the variance explained by other possible confounding variables in the data set: mixed effect modelling and Bayesian ANCOVA (see the ‘Methods’ section for details). Only results reaching threshold for both methods were considered robust enough to support our conclusions.

**Fig. 2.**
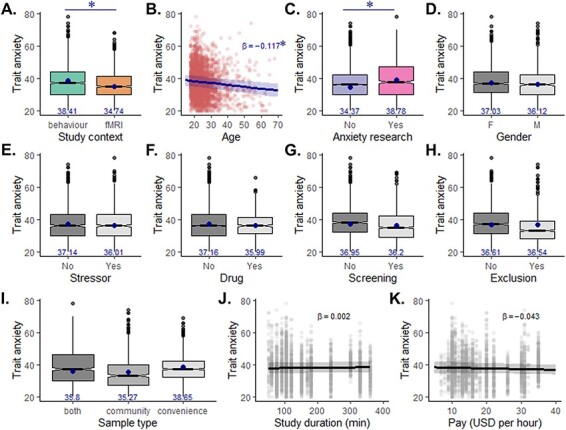
Main effects on trait anxiety (Model 1). A mixed effects model was run to predict trait anxiety scores from 11 variables, all competing for variance: (A) study context (behaviour *vs* fMRI), (B) age, (C) whether the study was part of anxiety research, (D) gender, whether the study involved (E) a stressor, (F) a drug administration procedure, (G) psychiatric screening, (H) whether data were provided after participant exclusion, (I) sample type, (J) study duration in minutes and (K) pay rate converted to USD per hour. Effects of categorical factors (A, C–I) are shown as box plots of the raw data; the blue dots and numbers represent the marginal means predicted from the model. Effects of continuous variables (B, J, K) are shown as scatter plots of trait anxiety as a function of the variable (dots: raw data; line: effect of the variable predicted by the model). The effects of study context, age and anxiety research (A–C) were found to be significant both in the mixed effects model and using Bayesian tests (*P < 0.001 and BF_10_ > 100).

In a linear mixed effects model (Model 1), we included fixed effects of all factors (study context, screening, gender, age, stressor, drug administration, sample type, study duration, pay rate, anxiety research and post-exclusion), as well as a fixed and random intercept for study site. We found a significant main effect of study context [estimate = −3.677 ± 0.43 (SE), χ^2^ = 71.29, *P* < 0.0001; Figure 2A], with an effect size over the difference in marginal means of *d* = 0.418 (averaged over the levels of all other factors). This indicates that lower trait anxiety in individuals participating in fMRI over behavioural studies is a robust effect in our large sample, present even when competing for variance with multiple other factors such as gender, age, study details and recruitment strategy. In fact, accounting for the variance explained by these variables yielded a 90% larger effect size. Bayesian analyses supported this finding, with the model including all main effects outperforming the same model lacking only the effect of the study context (BF_10_ > 10^14^). This is indicative of decisive evidence for this effect. While the size of the effect is variable across the specific study sites that provided data for both contexts (medium to large effect in Sites #1 and #9, small to medium effect in Sites #3 and #7 and negligible effect in Sites #5, #6 and #8; Table 3), trait anxiety in all the sites was numerically lower for the fMRI context.

### Higher trait anxiety in younger individuals and in studies focusing on anxiety research

We then set out to analyse the effect of other variables on trait anxiety to determine which effects are robust to the other variables in the model. The mixed effects model (Model 1) revealed significant effect of age [lower trait anxiety in older individuals: estimate = −0.117 ± 0.025 (SE), χ^2^ = 21.56, *P *< 0.0001; Figure 2B] and of anxiety research [higher trait anxiety in studies that are part of anxiety research: estimate = 4.416 ± 0.71 (SE), χ^2^ = 38.54, *P* < 0.001; Figure 2C]. Both were supported by the Bayesian test with decisive evidence (age: BF_10_ = 4528; anxiety research: BF_10_ > 10^7^). We note that the negative correlation between age and trait anxiety is robust to outliers (excluding individuals over age 50 years: *R*_3161_ = −0.136; excluding individuals over age 35 years: *R*_3001_ = −0.141; both *P* < 0.001).

Evidence for effects of gender, stressor and drug administration was mixed, as the mixed effects model suggested significant fixed effects [higher trait anxiety in females: estimate = −0.907 ± 0.32 (SE), χ^2^ = 7.773, *P* = 0.0053, Figure 2D; lower trait anxiety in studies involving a stressor: estimate = −1.137 ± 0.49 (SE), χ^2^ = 5.283, *P* = 0.022, Figure 2E; lower trait anxiety in studies involving drug administration: estimate = −1.171 ± 0.57 (SE), χ^2^ = 4.212, *P* = 0.04, Figure 2F]. However, the Bayesian analyses only indicated anecdotal evidence (gender: BF_10_ = 1.83; stressor: BF_10_ = 0.645, drug administration: BF_10_ = 0.392).

Finally, both analyses showed no significant effect of psychiatric screening [estimate = −0.747 ± 0.51 (SE), χ^2^ = 2.082, *P* = 0.149; Figure 2G], exclusion [estimate = −0.063 ± 0.66 (SE), χ^2^ = 0.009, *P* = 0.92, Figure 2H], sample type [community *vs* both estimates = −0.535 ± 0.69 (SE), convenience *vs* both estimates = 2.843 ± 1.73 (SE), χ^2^ = 3.327, *P* = 0.190; Figure 2I], study duration [estimate = 0.002 ± 0.001 (SE), χ^2^ = 1.785, *P* = 0.182, Figure 2J] and pay rate [estimate = −0.043 ± 0.033 (SE), χ^2^ = 1.435, *P* = 0.231, Figure 2K] on trait anxiety. Bayesian tests suggested strong to anecdotal evidence for these null effects (exclusion: BF_10_ = 0.075, sample type: BF_10_ = 0.18, screening: BF_10_ = 0.21, study duration: BF_10_ = 0.30, pay rate: BF_10_ = 0.51).

### Behaviour–fMRI trait anxiety differences are modulated by screening, drug administration and sample type

We then examined whether the difference in trait anxiety between behavioural and fMRI studies was moderated by any of the other variables. To test this, we built a second mixed effects model (Model 2) which, in addition to Model 1 effects, included the following two-way interactions with study context as fixed effects: context × gender, context × age, context × screening, context × stressor, context × drug, context × sample type, context × study duration and context × pay rate. A random effect of study context (with study site as random variable) was also included, allowing to model the interaction between context and site. Note that (i) this model only included the seven study sites that had data from both behavioural and fMRI study contexts, thus leading to a slightly reduced sample size of 3041, and (ii) given the small proportion of studies (especially fMRI studies) that were classified as anxiety research and provided data after participant exclusions, we were unable to include the interaction between study context and these variables. Finally, because the difference between behavioural and fMRI studies was our main question of interest, we did not investigate interactions between the other factors (i.e. not including study context).

**Fig. 3.**
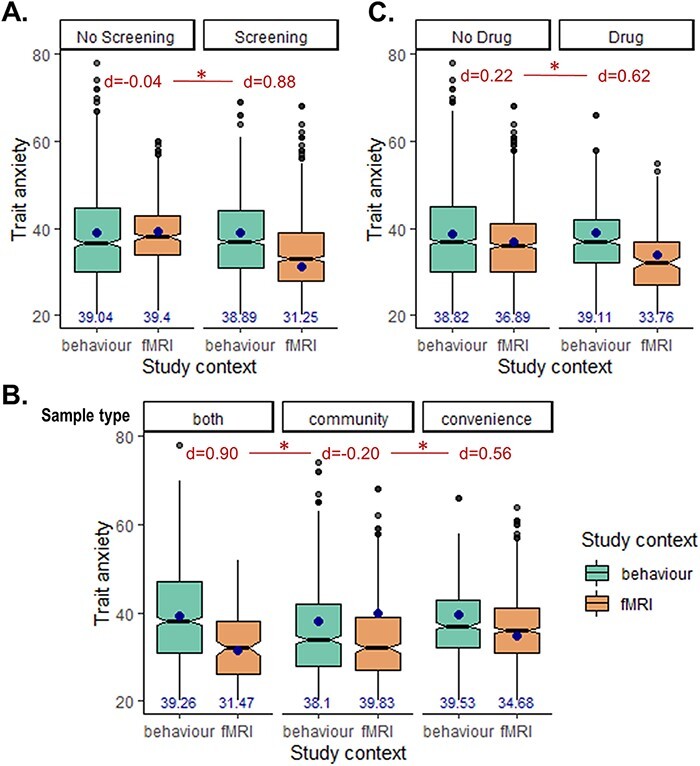
Moderating factors of the behaviour–fMRI difference in trait anxiety (Model 2). Interaction effects with study context were added to the mixed effects model, and the interactions with (A) screening, (B) sample type and (C) drug administration were found to be significant. Effects are shown as box plots of the raw data; the blue dots and numbers represent the marginal means from the interaction effect predicted by the model; the numbers in red represent the effect sizes associated with the behaviour–fMRI differences in marginal means.

We found significant interactions between the study context and screening [estimate = −8.008 ± 2.71 (SE), χ^2^ = 7.860, *P* = 0.005; Figure 3A], between study context and sample type [estimate = 9.525 ± 3.81 (SE), χ^2^ = 6.730, *P* = 0.035; Figure 3B] and between study context and drug administration [estimate = −3.414 ± 1.46 (SE), χ^2^ = 4.933, *P* = 0.026; Figure 3C]. All three interactions were supported by the Bayesian tests (context × screening: BF_10_ = 13.07; context × sample type: BF_10_ = 3.282; context × drug: BF_10_ = 4.742). The context × screening interaction was such that higher trait anxiety in behaviour compared with fMRI study contexts was only present when subjects were screened (effect size of difference in marginal means *d* = 0.88) compared with when they were not screened (*d* = −0.042). The context × sample type interaction was such that the behaviour–fMRI difference in anxiety was present in studies using convenience samples (*d* = 0.558) or a mix of convenience and community samples (*d* = 0.898) but not in studies relying on community samples only (*d* = −0.199). Finally, the context × drug interaction was such that the behaviour–fMRI difference in trait anxiety was larger in studies involving a drug administration procedure (*d* = 0.62) than in those without (*d* = 0.22). All other interactions were not significant (χ^2^ < 1.4, *P* > 0.24), as supported by the Bayesian tests (BF_10_ < 0.45). Given that some categories had no data (e.g. combined stress and drug administration study without screening), we refrained from investigating higher-level interactions than the ones reported above.

### Post-hoc analysis: effect of screening type

In the analyses reported above, participants were considered screened for affective/psychiatric disorders if either a phone screening or in-person structured interview was conducted and not screened if absence of psychiatric condition was based purely on self-report of meeting the eligibility criteria specified in the recruitment material or if no such eligibility criteria were specified. However, it is likely that the exact type of screening procedure (see Supplementary Table S2 for details) may differ across study contexts and play more of a modulatory role on trait anxiety scores. To examine this, we ran follow-up analyses in which instead of a binary variable, screening was classified into one of the three types: no screening, phone screening or full in-person screening. Numbers and mean trait anxiety for each screening type and study context are reported in Table 4, including the breakdown for those specific sites that used the same screening procedure across both study contexts. We found that the proportions of participants screened by phone, in person or not screened did not differ across study contexts (χ^2^ = 2.21, *P *= 0.33).

**Fig. 4.**
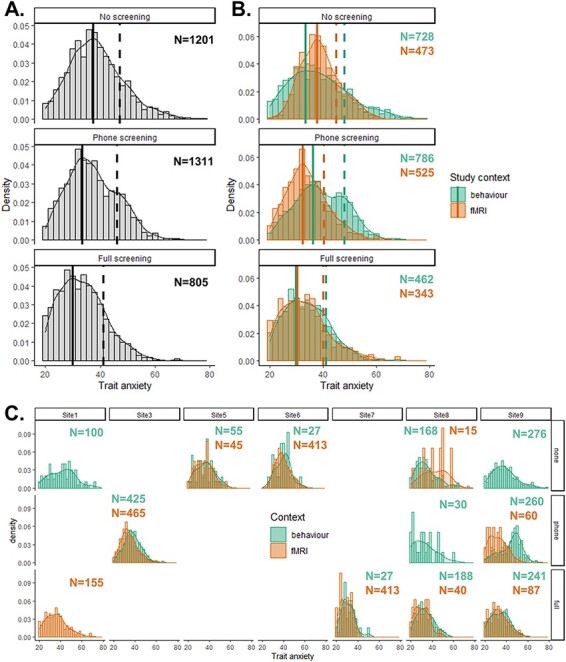
Distribution of trait anxiety scores split by screening procedure. Density plots of trait anxiety scores are shown (bin = 2), separately for individuals who were not screened for psychiatric/affective disorders (top panels), screened by phone (middle panels) or fully screened with an in-person structured clinical interview (bottom panels). Numbers of participants included in each distribution are shown above each density plot. (A) Distribution for the entire population. (B) Separate distributions for behaviour and fMRI study contexts. Solid lines in A and B show the mode of the distribution; dashed lines the 80th percentile. (C) Separate distributions across study contexts and across sites. Only sites that provided trait anxiety scores for at least one behavioural study and one fMRI study are included.

Re-running linear mixed effect Model 1, but distinguishing between phone and full screening procedures, showed that the difference in trait anxiety across study contexts remained significant [estimate = −3.388 ± 0.43 (SE), χ^2^ = 60.21, *P* < 0.0001, BF_10_ > 10^11^]. There was also a significant main effect of psychiatric screening type (χ^2^ = 41.24, *P* < 0.0001, BF_10_ > 10^6^, Figure 4A), with higher trait anxiety for unscreened compared with fully screened individuals [estimate = 2.653 ± 0.60 (SE)] and for individuals screened by phone compared with those that screened in person [estimate = 5.168 ± 0.82 (SE)]. Re-running linear mixed Model 2, testing for interactions with study context, revealed a significant interaction between the study context and the type of screening procedure (χ^2^ = 23.54, *P* < 0.0001, BF_10_ = 335.5). Mean trait anxiety scores collapsed across all sites (Table 4) showed that the interaction was driven by lower trait anxiety for fMRI relative to behaviour contexts when phone screening procedures were used (*T*_1245_ = 10.46, *P* < 0.001, *d* = 0.57) but not for studies with no screening (*T*_1198.7_ = −0.58, *P *= 0.56, *d* = 0.031) or studies with full in-person screening (*T*_688.74_ = 0.64, *P* = 0.52, *d* = 0.047).

Finally, examining the distribution of trait anxiety scores across study contexts and screening procedures (Figure 4B) revealed some interesting findings. First, while there was no difference in mean trait anxiety between behaviour and fMRI study contexts for unscreened individuals (Figure 4B top), the distributions exhibit several differences: the mode is lower for behavioural studies (33.43 *vs* 37.64), while the 80th percentile is lower for fMRI studies (45 *vs* 48), confirming the narrower distribution of trait anxiety scores in fMRI studies when no psychiatric screening is performed at recruitment. For individuals screened by phone (Figure 4B middle), both the mode (32.28 *vs* 36.15) and 80th percentile (40.2 *vs* 48) were lower in fMRI study contexts, driven by a smaller proportion of individuals scoring above 42. When individuals were fully screened using an in-person structured clinical interview (Figure 4B bottom), the two distributions matched almost exactly between study contexts (mode: behaviour = 29.72, fMRI = 30.40; 80th percentile: behaviour = 41, fMRI = 40).

We also note that specific sites could be driving some of the differences between no screening and phone screening (Table 4 and Figure 4C). Specifically, Site #9 made an important contribution to the difference observed in the case of phone screening (with Site #3 exhibiting a smaller but significant effect in the same direction). In the absence of screening, however, we see that Site #8 actually shows an effect in the opposite direction, with the caveat that the sample size for the fMRI group in Site #8 is extremely small (*N* = 15), making the comparison for this particular site very underpowered and difficult to interpret. Overall, this heterogeneity between sites seems reduced in the case of full screening, for which trait anxiety scores are consistent across study contexts in all three sites that provided data for this arm (i.e. Sites #7, #8 and #9).

### Relationship with state anxiety

**Fig. 5.**
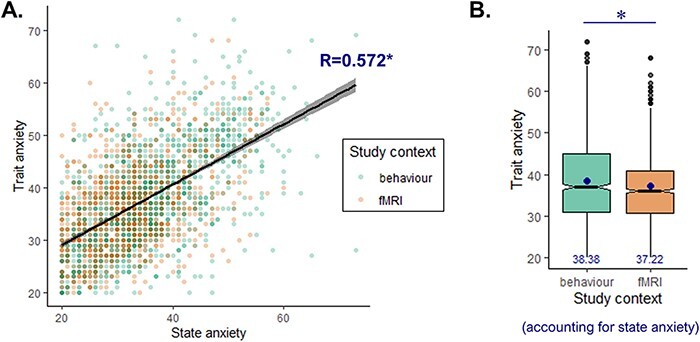
Dissociating state and trait anxiety. (A) We obtained state anxiety scores in a subset of the data (*N* = 2324) and plot trait anxiety as a function of state anxiety for each of these individuals, as well as the best-fitting regression line. (B) The difference in trait anxiety between behavioural and fMRI studies remains significant when regressing out the variance explained by state anxiety.

Given that trait and state anxiety scores are generally highly correlated, it is possible that the observed difference in trait anxiety between behavioural and fMRI studies is in fact driven by state anxiety scores. To assess this possibility, we gathered state anxiety scores for a subset of the entire data set (*N* = 2324) across five sites with both behavioural and fMRI studies (Supplementary Figure S1). The correlation between state and trait anxiety in these individuals was indeed high (*R* = 0.572, *P *< 0.001, Figure 5A), and there was a significant difference in state anxiety between study contexts (*T*_2289.6_ = 9.59, *P* < 0.001, Supplementary Figure S1B). Nonetheless, the amount of unshared variance between the two variables (67.3%) was sufficient to examine the effect of study context on trait anxiety while regressing out the variance explained by state anxiety scores. To do so, we ran a final mixed effects model, which predicted trait anxiety from study context and state anxiety (see the ‘Methods’ section for details). We found that the effect of study context on trait anxiety remained significant [estimate = −1.162 ± 0.39 (SE), χ^2^ = 8.848, *P* = 0.003, BF_10_ = 6.04; Figure 5B] and thus could not be explained by state anxiety alone.

## Discussion

In this study, we provide substantial evidence, in a large-scale data set of healthy participants across multiple sites, that individuals participating in fMRI studies exhibit on average lower levels of trait anxiety than individuals participating in behavioural studies only. We show that this effect is even stronger when regressing out the variance in trait anxiety explained by multiple other factors, such as age, gender, but also recruitment strategies (sample type, psychiatric screening) and other study details (presence of a stressor or drug, study duration, pay rate and anxiety research). In addition, the trait anxiety difference remained significant when state anxiety scores were included in the model. Both mixed modelling approaches and Bayesian analyses supported this effect. Interestingly, while the mean difference in trait anxiety scores was relatively small (2-point difference, effect size = 0.219), we note that the effect size nearly doubled (4-point difference in the marginal means, *d* = 0.418, Figure 2A) when variance due to other factors was regressed out, indicative of a moderate effect size. Additionally and importantly, distributions across study contexts markedly differed from each other, with a much narrower and somewhat truncated distribution of trait anxiety scores in fMRI studies relative to behavioural studies. A recent study reported similar distributions of trait anxiety scores when comparing their behavioural and fMRI samples (Sjouwerman *et al.*, 2020). This difference in the distributions has two main consequences. First, it suggests that non-clinical fMRI samples are less representative of the general population than non-clinical behavioural samples. Second, the narrower range will make examining individual differences in trait anxiety more difficult in fMRI compared with behavioural studies.

Procedures in place to screen participants for psychiatric and/or affective disorders were found to modulate the distribution of trait anxiety scores in different ways for fMRI and behavioural study contexts. Specifically, when no or minimal (i.e. phone) screening was performed, the range of trait anxiety scores was narrower in fMRI compared with behavioural studies, while the two distributions matched when full in-person clinical interviews were used. Several possible factors could explain the observed differences. For studies using phone or online screening procedures, participants with higher anxiety might be screened out of fMRI studies more often than behavioural studies, because of inherent differences in screening questions. For example, during recruitment of fMRI studies, participants are likely asked additional screening questions, such as history of claustrophobia, which would usually not be asked for behavioural studies. It is also possible that participants are more likely to not reveal, or not be aware of, their history of psychiatric disorders when participating in a behavioural study for which screening does not occur in person. Finally, a self-selection bias during recruitment is also possible, whereby individuals with high trait anxiety are less likely to volunteer to participate in fMRI studies, even if they meet all eligibility criteria. When full in-person clinical screening is performed, however, we believe that participants with higher anxiety are more likely to be excluded from the study, given the high comorbidity between elevated anxiety and many disorders from the Diagnostic and Statistical Manual of Mental Disorders (DSM-5), for which meeting criteria will usually result in exclusion from a healthy control sample. This is irrespective of whether the study involves neuroimaging or not. Undergoing MRI scanning has been found to be anxiogenic, because of claustrophobia, discomfort and/or fear of learning about potential incidental findings (Meléndez and McCrank, 1993; Katz *et al.*, 1994; Murphy and Brunberg, 1997); therefore, anxious individuals are likely to find the experience of MRI scanning more aversive and elect not to participate. While excluding participants with claustrophobia from fMRI studies may partly explain the bias (Katz *et al.*, 1994; Murphy and Brunberg, 1997), whether other specific components of anxiety play a role remains unclear. Factor analyses of the STAI (Bieling *et al.*, 1998; Vigneau and Cormier, 2008; Wang *et al.*, 2018) suggested different components of trait anxiety, such as anxiety-present *vs* anxiety-absent components (corresponding to items reflecting negative *vs* positive emotional experiences) or components assessing anxiety, worry, sadness, self-deprecation as well as general negative affect. Whether a subset of these components is more likely to weigh in on the decision to take part in an fMRI or behavioural study remains an open question for future investigation. We note this analysis was beyond the scope of the present study, given that individual item scores from the trait anxiety questionnaires were not obtained in the data.

Examining the distributions of trait anxiety scores across sites, screening procedures and study context (Figure 4C) indicates substantial between-sites heterogeneity in how screening procedures may modulate the behaviour–fMRI trait anxiety difference. This raises the possibility that there is still a lot of unexplained variance between sites. Such heterogeneity is likely due to the observational nature of the study—analysing existing data rather than carefully controlling variables between sites to allow for robust comparisons and quantification of interaction effects. Therefore, site differences should be interpreted with caution since any inferred cause for these differences is likely to be speculative. Instead, we hope that future studies will rely on carefully controlled designs or experimental manipulations to empirically address whether and how participants’ decisions to sign up for a study and researchers’ decisions to include participants are influenced by the screening procedure, specific questions asked during screening, the recruitment materials or the participant’s level of anxiety during sign-up. This would allow determining whether the sampling bias arises before or after screening. Nevertheless, the present findings are important and robust to those site-specific effects since our regression model accounts for variance between sites, suggesting that trait anxiety is lower in fMRI compared with behavioural studies over and beyond the differences observed between sites.

Our results also revealed that the type of sample or participant pool subjects are recruited from seems to matter, consistent with previous evidence suggesting an effect of sample composition on neuroimaging findings (LeWinn *et al.*, 2017). Specifically, the difference in trait anxiety between fMRI and behavioural studies was larger in studies that relied at least partly on convenience samples than in studies recruiting from the community. Finally, the effect was also larger in studies involving a drug administration procedure (i.e. pharmacology studies), suggesting that the sampling or self-selection bias towards individuals with low trait anxiety is more evident in studies combining fMRI with pharmacology (compared with fMRI only). Furthermore, our findings speak of other factors that explain some of the variance in individual trait anxiety scores. We found a negative correlation between age and trait anxiety, consistent with past literature suggesting trait anxiety decreases with age (Knight *et al.*, 1983; Nakazato and Shimonaka, 1989; Regier *et al.*, 1990). The evidence for an effect of gender on trait anxiety, however, was mixed. Consistent with the literature suggesting both higher prevalence of anxiety disorders (McLean *et al.*, 2011) and higher self-reported anxiety (Knight *et al.*, 1983; Spitzer *et al.*, 2006) in females than males, we also report higher trait anxiety in females. This effect was significant in the mixed effects model but was not robustly supported by Bayesian tests. We also found higher trait anxiety in studies that were considered part of anxiety research, possibly because these studies might mention their relevance to anxiety research in recruitment materials and therefore be more likely to appeal to participants experiencing more anxiety.

While the large scale of the present data set allowed us to ensure the robustness of the effects, with data obtained from multiple institutions and regressing out the effects of multiple potential confounds, we note possible limitations. First, contributing institutions were mostly located in the USA and northern Europe, thus leaving open the possibility that the observed effects may not generalize to data collected in other parts of the world. Second, the variables we included in the analyses (age, gender, screening type, sample type, stressor, drug, study duration, pay rate, study site, anxiety research and exclusion) are of course not exhaustive, and one could imagine that other mediators are likely to explain additional variance in trait anxiety scores and/or in the willingness to participate in fMRI studies (Leach *et al.*, 2008). Examples include socioeconomic status, race/ethnicity, urban living, ruminative and depressive states, neuroticism, physical health, remuneration or other components of the study design. Collecting these additional variables would not have been possible in the current data set, given that they were either not measured in the first place or would have compromised the anonymization of the data set. Finally, we found the difference in trait anxiety scores was found to be robust to state anxiety in a subset of the data; however, we do not discard a possible role of state anxiety in the self-selection bias as well. Similarly, recent literature suggests that trait anxiety may not exclusively measure anxiety *per se* but rather reflect negative affect more generally (Hur *et al.*, 2019; Knowles and Olatunji, 2020), both in its behavioural and neurobiological signatures (Shackman *et al.*, 2016). Whether the difference observed between fMRI and behavioural participants is specific to trait anxiety or relates to general negative affect thus remains an open question.

Overall, the finding of lower trait anxiety, as well as narrower distribution of trait anxiety scores, in fMRI compared with behavioural studies has implications for both previously published and future research in the field of cognitive neuroscience as a whole and for anxiety research more specifically. These differences may be responsible for failed replications, whereby a behavioural effect of interest, and/or a moderating effect of trait (or induced/state) anxiety, evidenced in a behavioural study, fails to replicate in a follow-up fMRI study (e.g Bolton and Robinson, 2017; Garibbo *et al.*, 2019) or vice versa. Because of the narrower range of trait anxiety values in fMRI studies, this may also enhance the differences between patient and control groups in studies of psychiatric populations, whereby control subjects have lower trait anxiety than the general population. While the present findings may carry some relevance for clinical studies, we note that the data set did not include any trait anxiety scores from clinically anxious individuals; therefore, we do not know whether the observed difference between behavioural and fMRI study contexts would expand to clinical samples. It is possible that the bias may not actually be present in clinical studies, given that patients’ motivations or benefits for participating in research may be different and lead to greater recruitment into fMRI studies than in non-clinical samples. This intriguing possibility warrants further investigation.

Taken together, these findings point towards possible recommendations for cognitive neuroscience researchers who run both fMRI and behavioural studies to measure individual differences in anxiety and carefully consider and mitigate potential sources of recruitment bias. Our finding that distributions of trait anxiety scores between fMRI and behavioural studies match almost perfectly when full in-person psychiatric screening interviews are conducted suggests that such screening procedures may be one way to ensure similar levels of trait anxiety across study contexts. This is particularly relevant for researchers recruiting from convenience samples (i.e. undergraduate students) or running pharmacology studies, both of which were associated with larger trait anxiety differences between behavioural and fMRI contexts. However, while screening can ensure better matching of trait anxiety across study contexts, it may still lead to samples that are not representative of the general population, as anxious individuals may be excluded from all study contexts at a higher rate. This could result in a loss of power to examine individual differences and undermine relevance for psychopathology.

A solution would then be to rely on methods that help recruit participants with higher anxiety into fMRI studies, such as the use of a mock scanner, virtual reality or psychological interventions. These methods have been successful in alleviating MRI anxiety in paediatric populations (Viggiano *et al.*, 2015) and patients with anxiety (Garcia-Palacios *et al.*, 2007; Tugwell *et al.*, 2018) and could therefore be expanded to the general population to reduce sampling biases. Using stratified sampling, whereby trait anxiety or dispositional negativity is measured at screening in a large sample after which study participants are selectively recruited from that sample to ensure representation across the full range, would also help mitigating the observed bias (Hur *et al.*, 2020).

In conclusion, our recommendations in the light of the present findings are as follows. First, regardless of the specific causes behind this bias, this study sheds light on the possibility that fMRI samples are less representative of the general population than behavioural samples, or at the very least that behavioural and fMRI samples are different from each other, when it comes to trait anxiety. This is likely to be problematic for mechanistic or experimental research, irrespective of generalizability. Second, researchers should adopt recruitment and/or screening strategies that can help them mitigate this bias if it is likely to impact the validity or interpretation of their results. Finally, future research should further explore possible causes of this bias and mitigation strategies, particularly through controlled experiments. Probing more deeply into individual reasons for participating in fMRI studies and differences in screening procedures seems necessary to ensure researchers can enforce a distribution of psychological and psychiatric profiles that is representative of the general population.

## Supplementary Material

nsab057_Supp
