# Supplementary material for: How representative are neuroimaging samples? Large-scale evidence for trait anxiety differences between fMRI and behaviour-only research participants
Source: Soc Cogn Affect Neurosci. 2021 May 5;16(10):1057–70. doi: 10.1093/scan/nsab057 (PMC8483285; doi:10.1093/scan/nsab057)
Supplement: nsab057_Supp [file nsab057_supp.zip › nsab057_suppl.pdf]

Supplementary Information

How representative are neuroimaging samples? Large-scale evidence for trait anxiety differences between fMRI and behaviour-only research participants.

Caroline J. Charpentier[1]*, Paul Faulkner[2], Eva R. Pool[3], Verena Ly[4], Marieke S. Tollenaar[4], Lisa M. Kluen[1], Aniek Fransen[1], Yumeya Yamamori[5], Níall Lally[5], Anahit Mkrtchian[5], Vincent Valton[5], Quentin J.M. Huys[5], Ioannis Sarigiannidis[5], Kelly A. Morrow[6], Valentina Krenz[7], Felix Kalbe[7], Anna Cremer[7], Gundula Zerbes[7], Franziska M. Kausche[7], Nadine Wanke[7], Alessio Giarrizzo[3], Erdem Pulcu[8], Susannah Murphy[8,9], Alexander Kaltenboeck[8,10], Michael Browning[8,9], Lynn K. Paul[1], Roshan Cools[11,12], Karin Roelofs[11], Luiz Pessoa[6], Catherine J. Harmer[8,9], Henry W. Chase[13], Christian Grillon[14], Lars Schwabe[7], Jonathan P. Roiser[5], Oliver J. Robinson[5], John P. O'Doherty[1]

[1]California Institute of Technology, Pasadena, CA, USA. [2]University of Roehampton, London, UK. [3]University of Geneva, Geneva, Switzerland. [4]Dept of Clinical Psychology, Leiden University; Leiden Institute for Brain and Cognition, Leiden, the Netherlands. [5]Institute of Cognitive Neuroscience, University College London, London, UK. [6]University of Maryland, College Park, MD, USA. [7]University of Hamburg, Hamburg, Germany. [8]Dept of Psychiatry, University of Oxford, Oxford, UK. [9]Oxford Health NHS Trust, Oxford, UK. [10]Dept of Psychiatry and Psychotherapy, Clinical Division of Social Psychiatry, Medical University of Vienna, Austria. [11]Donders Institute for Brain Cognition and Behaviour, Radboud University, Nijmegen, the Netherlands. [12]Dept of Psychiatry, Radboud University Medical Centre, Nijmegen, the Netherlands. [13]Dept of Psychiatry, University of Pittsburgh, Pittsburgh, PA, USA. [14]National Institute of Mental Health, Bethesda, MD, USA.

*Corresponding author contact information: ccharpen@caltech.edu

Site	Study context	Recruitment from past studies	Sample type (community, convenience, both)	Anxiety research	All subjects included	MRI-naive subjects only	Mock scanner
#1	**Behaviour**	unknown	community	no	no		
	**fMRI**	unknown	community	no	yes	no	no
#3	**Behaviour**	partly	convenience	no	73% yes, 27% no		
	**fMRI**	partly	convenience	no	yes	no	no
#5	**Behaviour**	partly	convenience	no	yes		
	**fMRI**	partly	convenience	yes	yes	no	no
#6	**Behaviour**	unknown	convenience	no	yes		
	**fMRI**	unknown	convenience	no	yes	no	no
#7	**Behaviour**	75% no, 25% yes	community	yes	no		
	**fMRI**	75% no, 25% yes	community	yes	no	no	no
#8	**Behaviour**	no	62% community only, 38% both	8% yes, 92% no	8% yes, 92% no		
	**fMRI**	no	community	no	27% yes, 73% no	no	no
#9	**Behaviour**	75% unknown, 25% no	11% community only, 89% both	55% yes, 45% no	yes		
	**fMRI**	73% unknown, 27% no	6% community only, 94% both	50% yes, 50% no	61% yes, 39% no	no	no

Table S1. Recruitment and inclusion strategies across sites and across study contexts within sites. The following study-level characteristics are shown for each site that provided data for both study contexts: whether participants had taken part in previous studies, whether participants were recruited from community sample, convenience sample (i.e. undergraduate students), or a mix of both, whether the study was part of anxiety research, whether trait anxiety data from all recruited participants was included in the dataset (or only data post-exclusions), and for fMRI studies, whether only MRI-naive subjects were recruited and whether participants were exposed to a mock scanner.

	Behaviour		fMRI	
	**N**	**Screening procedure**	**N**	**Screening procedure**
Site #1	100	**None:** study flyer specifying no psychiatric disorder	155	**Full:** MINI
Site #2	102	**None:** no exclusion criteria for psychiatric or neurological disorders	0	-
Site #3	425	**Phone:** smoking, medication intake, drug use, history of neurological/ psychiatric diagnosis, history of drug abuse.	465	**Phone:** same interview as Behaviour
Site #4	71	**Phone:** BMI, medication use, alcohol use, smoking, drug use, severe illness in past year, diabetes, psychiatric problems in past year requiring therapy, follow-up MDD symptoms	0	-
Site #5	55	**None:** self-report of meeting eligibility criteria stated on information letter – no neurological, cardiovascular diseases, psychiatric disorders, regular use of medication or marijuana, use of psychotropic drugs	45	**None:** same as Behaviour + claustrophobia & heavy smoking
Site #6	27	**None:** self-report of meeting eligibility requirements – no psychoactive drugs, no psychological conditions, no neurological condition	413	**None:** same as Behaviour
Site #7	33	**Full:** SCID	61	**Full:** SCID
Site #8	188	**Full:** SCID	40	**Full:** SCID
	30	**Phone:** exclude past history of psychiatric or neurological disorder		
	168	**None:** self-report of no use of psychotropic medication, no diagnosis of current Axis I disorder, BMI between 18 and 30	15	**None:** same as Behaviour
Site #9	241	**Full:** MINI	87	**Full:** MINI
	260	**Phone:** past/present diagnosis of depression, stress-related problems, bipolar disorder, ADHD, eating disorder, OCD, trichotillomania, learning disability, alcohol & drug abuse, medication for psychiatric disorder	60	**Phone:** same interview as Behaviour + claustrophobia
	276	**None:** self-report of meeting eligibility criteria specified on study advert – no past or present psychiatric or neurological disorder, alcohol use (past 24hrs) or cannabis (past week).		

Table S2. Details of screening procedures across study sites and study contexts. The number of individual data points for each site and each study context are reported, as well as details about the screening procedure and eligibility criteria. SCID: Structured Clinical Interview for DSM-5 Axis I disorders. MINI: Mini-International Neuropsychiatric Interview. BMI: Body-Mass Index. MDD: Major Depressive Disorder. ADHD: Attention Deficit Hyperactivity Disorder. OCD: Obsessive Compulsive Disorder.

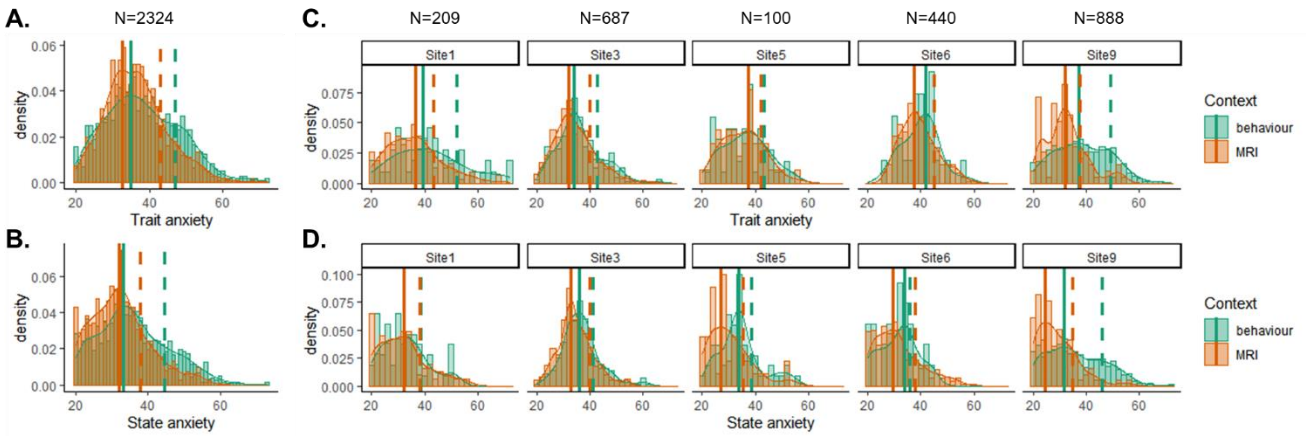

Figure S1. Comparing trait and state anxiety distributions. State anxiety scores were obtained for a subset of the data (N=2324) across 5 study sites that provided data for both behavioural and fMRI participants. (**A-B**) Distribution of trait (**A**) and state (**B**) anxiety scores collapsed across sites. (**C-D**) Distribution of trait (**C**) and state (**D**) anxiety scores split per site. Solid lines show the mode of the distribution; dashed lines the 80th percentile.
